# Supplementary material for: Molecular Simulation of the Binding of Amyloid Beta to Apolipoprotein A-I in High-Density Lipoproteins
Source: Int J Mol Sci. 2025 Feb 6;26(3):1380. doi: 10.3390/ijms26031380 (PMC11818119; doi:10.3390/ijms26031380)
Supplement: Supplementary file 1 [file ijms-26-01380-s001.zip › ijms-3407343-supplementary.pdf]

Molecular Simulation of the Binding of Amyloid Beta to Apolipoprotein A-I in High-Density Lipoproteins

Chris J. Malajczuk and Ricardo L. Mancera

Supplementary Materials

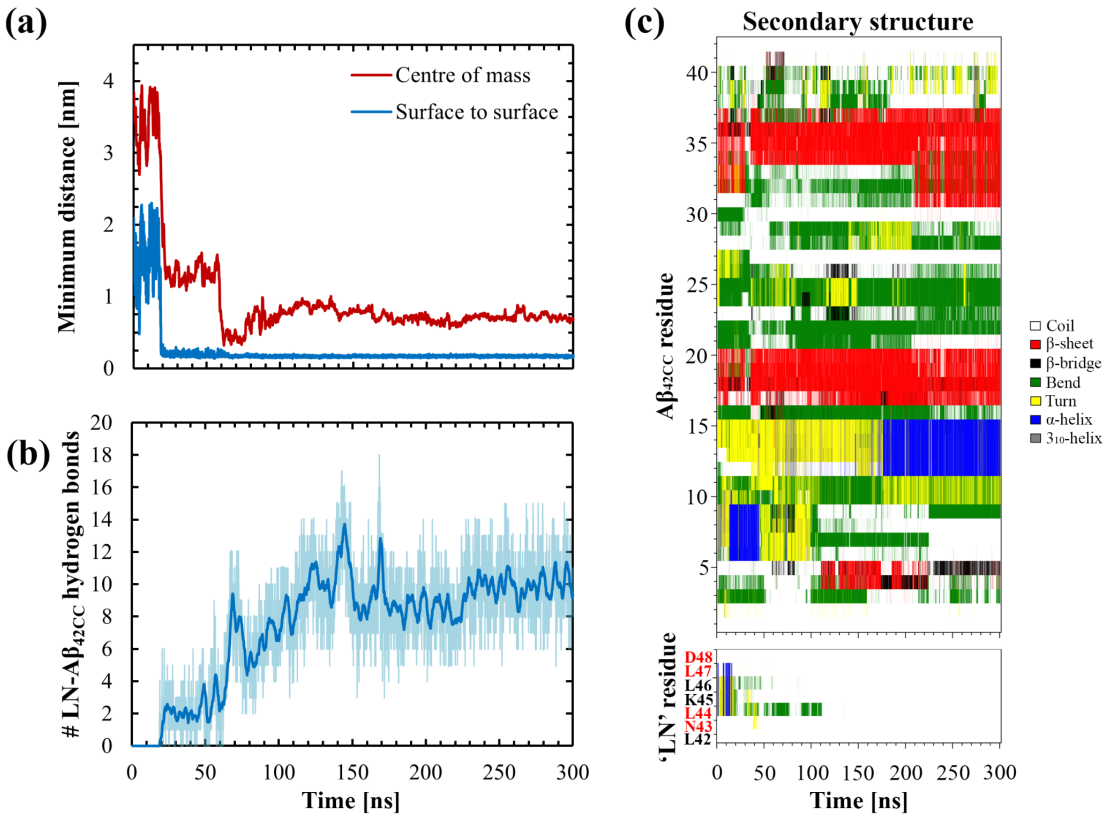

Figure S1. (a) Minimum intermolecular distances between Aβ_{42CC} and 'LN' as a function of conventional MD simulation time. (b) The time-wise number of intermolecular hydrogen bonds formed between Aβ_{42CC} and 'LN' in a conventional MD simulation. (c) The secondary structures sampled across the Aβ_{42CC} peptide (top) and 'LN' fragment (bottom) during a conventional MD simulation.

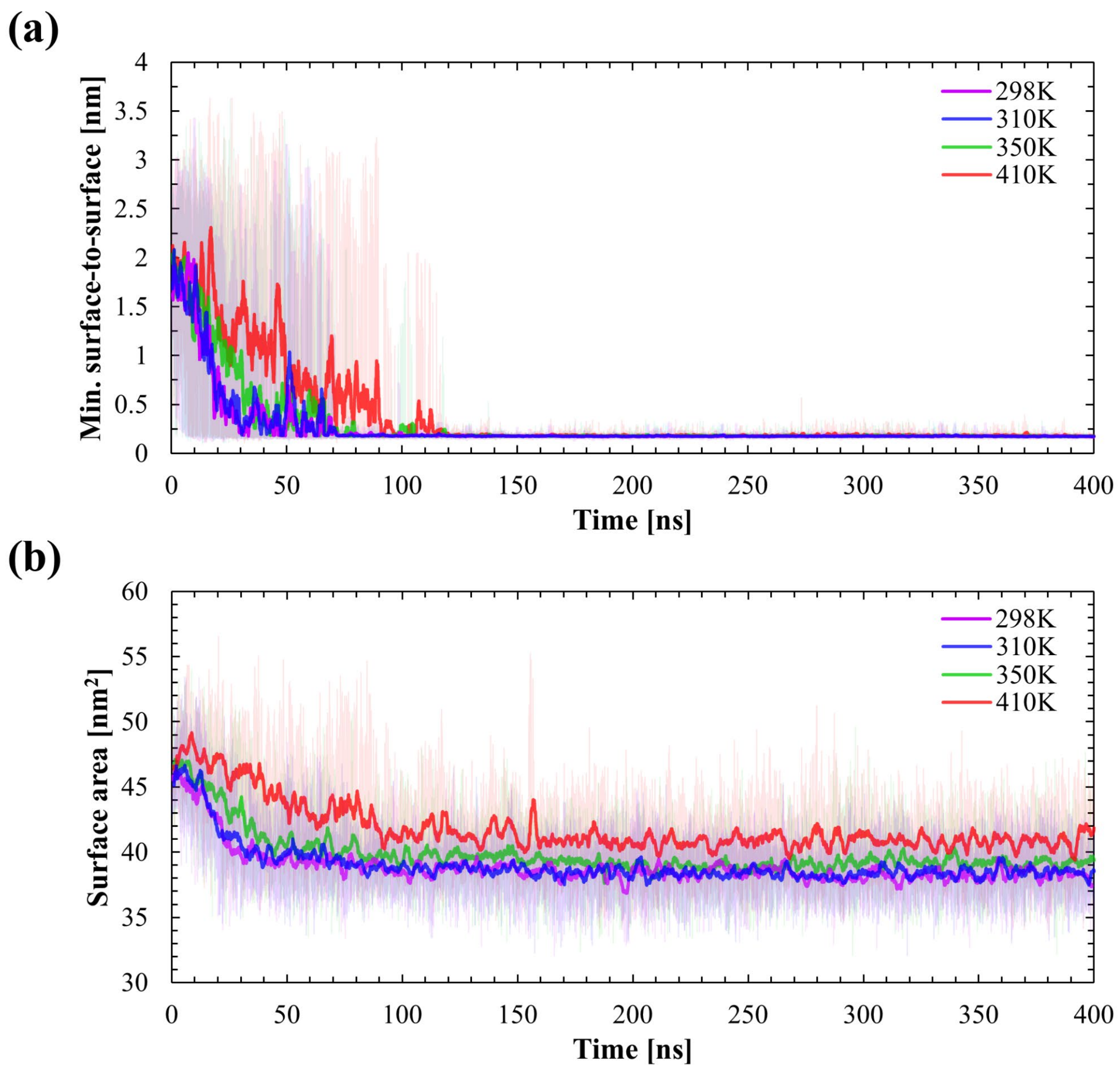

Figure S2. (a) Minimum surface-to-surface distance between the 'LN' fragment and the Aβ_{42CC} peptide at four representative temperatures across the T-REMD simulation. According to this metric, complexation of Aβ_{42CC} and 'LN' proceeded across the entire T-REMD temperature range. (b) Combined total complex surface area for REMD simulations of the 'LN' fragment and the Aβ_{42CC} peptide at four representative temperatures across the T-REMD simulation duration. In each panel, the instantaneous values are plotted as transparent lines in the background, whilst running averages across a 0.5 ns window are plotted as opaque lines in the foreground.

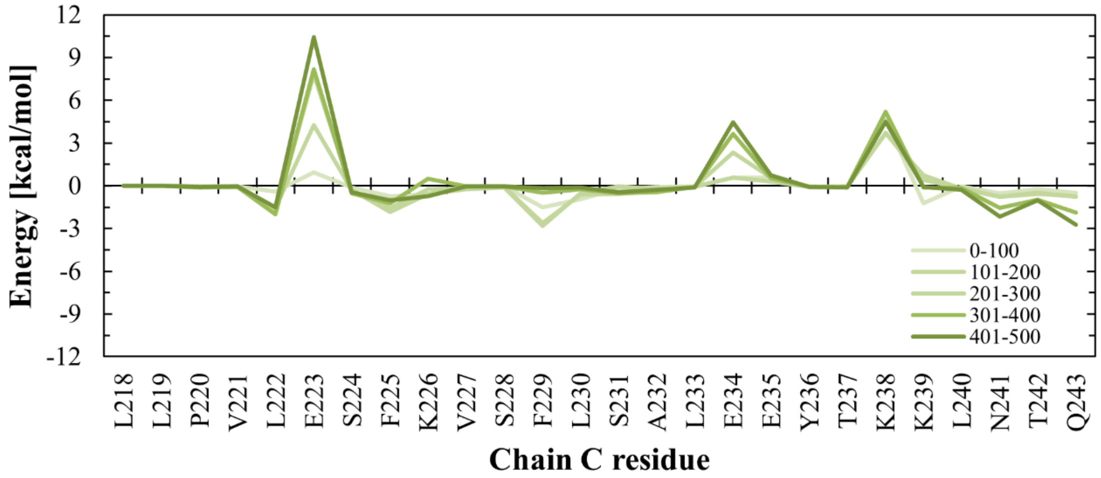

Figure S3. Per-residue decomposition of binding free energies across the final 25 residues of an apoA-I chain bound to Aβ_{42CC} via its 'LN' segment. Throughout the simulation, three charged residues in this segment of apoA-I (E223, E234 and K238) exerted a significant positive contribution to the overall binding free energy due to the decoupling of stabilising salt-bridges within a trefoil arrangement in preference for the formation of transient interactions with Aβ_{42CC}.
